# Empowering Elementary and Middle School Youth to Speak Up and Be Safe: Advancing Prevention of Child Maltreatment with a Universal School-Based Curriculum

**DOI:** 10.3390/ijerph191911856

**Published:** 2022-09-20

**Authors:** Wendy Wolfersteig, Marisol Juarez Diaz, Diane Moreland

**Affiliations:** Southwest Interdisciplinary Research Center (SIRC), School of Social Work, Watts College of Public Service and Community Solutions, Arizona State University, Phoenix, AZ 85004, USA

**Keywords:** child maltreatment, prevention, child abuse and neglect, evidence-based intervention, school safety curriculum, universal prevention program, bullying

## Abstract

Child maltreatment is a global public health problem, and school-based universal prevention programs such as the Speak Up Be Safe (SUBS) curriculum can be an effective solution to help address child violence. This randomized control study employed a pre-, post-, and six-month follow-up design for students in kindergarten to grade 8, approximately ages 5–13 (*n* = 2797). Surveys measured the efficacy of the curriculum in increasing students’ knowledge of safety rules and self-protection strategies. The analyses explored the differences at follow-up between the conditions for each index/scale for each grade using an analysis of covariance, which controlled for the pre-survey scores. The SUBS group had significantly higher scores at follow-up than the students in the control group. This study showed that the students learned new knowledge and skills to act upon and identify child abuse and neglect in keeping themselves and others safe. Policy- and decision makers now know that as a child maltreatment prevention program, SUBS can be implemented universally in schools at a low cost, delivering an essential evidence-based safety curriculum that protects students from child maltreatment.

## 1. Introduction

Child maltreatment (CM) is an international public health concern that generates both short-term harm and long-lasting negative effects, such as physical, behavioral, and psychological problems, and substantially contributes to child death, injury, and disability [1]. CM comprises various forms of offending against children including physical and sexual abuse, neglect, and emotional violence (i.e., bullying) [2]. Neglect, CM, and unusually stressful or traumatic conditions contribute to adolescents’ risky behaviors and their development of maladaptive coping mechanisms [3,4,5]. According to the World Health Organization, an estimated one out of two children (aged 2–17 years), or one billion children, suffer some form of violence each year [6,7]. More specifically, 120 million girls worldwide are estimated to have suffered some form of forced sexual contact before the age of 20, and [8,9] emotional violence affects one in three children, with a third of students aged 11–15 years having been bullied by their peers in the past month [10,11].

Furthermore, child protection continues to be drastically impacted by the pandemic. The onset of COVID-19 as a new public health crisis has magnified adverse conditions for children, families, and communities that are costly to our society. For instance, scholars provide evidence that lockdowns may have increased children’s vulnerability to maltreatment at home due to heightened poverty, food and housing insecurity, parental unemployment, intimate partner violence, and systemic inequalities [12]. In addition, developing research reports that there were adverse impacts of lockdowns on the system’s capacity to see, hear, and protect children from abuse [13,14]. The various increased CM risk factors pave a trajectory for adverse health outcomes that are of great concern, as children’s health-related status can influence their physical and mental health as adults [15,16,17]. Additionally, more research is examining how community-wide trauma impacts CM risk, specifically around how COVID-19 created mandated physical separation and intensified fears, which could result in delayed medical care for children [18,19].

Universal prevention strategies are critical to ensuring the safety of our children, and a growing response to address CM is through school-based prevention programs [20,21]. Universal prevention education considers all settings, including family, school, and community, and can help define healthy behaviors. More specifically, the school-based prevention model has proven to be effective in addressing various adolescent issues such as alcohol use, pregnancy prevention, mental health promotion, and bullying [22,23,24]. In addition to improving students’ knowledge of personal safety skills, school-based curricula have been found to increase disclosure, decrease self-blame, and reduce the rates of future victimization [25].

Moreover, school-based curricula are well-suited for CM prevention delivery because programs can be implemented universally at comparatively little cost, and the content aligns with school health curricula [26] or other programs focused on student well-being such as Social Emotional Learning (SEL). SEL programs are employed in schools to respond to the mental well-being and safety concerns of students [27]. It would be extraordinary to see students gain the benefit of both SEL and CM prevention curriculums, which are reported to have positive behavioral and academic benefits [28].

Besides the abovementioned advantages, teachers, counselors, and school staff are in an optimal position to prevent, identify, and assist because of their frequent contact with students and their role as mandated reporters. Over 90% of children are in primary school [29] where elementary-age children are at substantial risk for CM [30]. Offering primary prevention programs in schools allows for all children to understand as well as recognize abuse, teaches the skills that decrease children’s risk for abuse, and provides a positive space for disclosure. Furthermore, in addition to educating the students, the programs usually include training and resources for the parents and school officials.

School-based CM programs are most effective when delivered to all ages with racially multicultural groups [31]. However, there is a shortage of evidence-based and developmentally appropriate programs available to schools [32]. Thus far, most prevention programs exclusively focus on preventing child sexual abuse (CSA). Additionally, evaluations often lack rigor and diversity, and few studies evaluated the long-term retention of knowledge [33]. Del Campo and Fávero reviewed 70 evaluations of CSA prevention programs and found that only 17 studies carried out a follow-up study that exceeded two months [34]. This lack of follow-up has impacted the ability to compare curricula and outcomes and has brought requests from policymakers, researchers, educators, and communities for more rigorous research, including evidence-based CM prevention programs.

Recognizing that there are a limited number of evidence-based and developmentally appropriate CM curricula created for elementary and middle school students, this study contributes to the literature. This paper reports the findings of a randomized controlled trial testing the effects of a universal school-based prevention curriculum, Childhelp’s Speak Up Be Safe (SUBS) curriculum. The SUBS curriculum is designed to help youth of all ages learn the skills and strategies that enhance personal safety as well as emphasize the role of adults in keeping children safe [35]. For this study, a pre-, post-, and six-month follow-up quantitative design was adopted for students in kindergarten to grade 8 (approximately ages 5–13). Surveys were used to measure the efficacy of the SUBS curriculum in teaching the knowledge of safety rules and self-protection strategies.

### 1.1. Speak Up Be Safe (SUBS) Curriculum

Childhelp’s SUBS curriculum is designed for all grades (pre-kindergarten to 12) and focuses on disrupting all forms of child maltreatment, including neglect and online bullying. The SUBS curriculum teaches age-appropriate definitions and universal strategies to foster self-protective skills with developmentally appropriate materials [36] that are grounded in ecological system theory [37] and resiliency theory [38,39]. Each SUBS lesson (at every grade level) includes activities to provide practice on the application of skills across multiple contexts to distinguish what students need to stay safe and healthy. The SUBS content and delivery were researched based on several topic areas, and each lesson at every grade level is structured to reflect the principles of learning theory [40].

The SUBS curriculum was developed to be facilitated by school social workers, counselors, or teachers for two 30 to 45 min sessions at each grade level, with approximately 25–30 students. The overarching goal was to teach children the skills and strategies to prevent, interrupt, and seek help from safe adults when faced with abuse. The SUBS curriculum is aided through presentation and discussion materials that are available through an online platform. The lessons are to be delivered several weeks apart, with flexibility for school schedules. The curriculum manual also includes the big ideas for each class to ensure that age-appropriate messages are taught and aligned from grade to grade; the manual also defines students’ developmental characteristics at each grade level.

Students learn how to be safe through various concepts, key terms, and safety rules, depending on their grade level. Students in kindergarten through grade 2 learn safety knowledge. Starting in grade 3, students learn the SUBS five safety rules: (1) *It’s my Body*!; (2) *Ask an adult if I am safe*; (3) *I have choices*; (4) *Tell someone*, and (5) *It’s never my fault!* Students in grades 4 through 8, in addition to these safety rules, also learn six resistance strategies using the acronym RESIST (*Run*, *Escape*, *Scream*, *Ignore*, *Stay Away*, and *Tell*). Accordingly, each lesson offers activities to tap students’ prior knowledge, promote social interaction, engage them in various learning strategies, and provide practice focused on applying skills and techniques across multiple contexts. A key message of SUBS is for youth to *Speak Up* when they encounter abuse or see someone else experiencing CM; thus, SUBS can be a safety tool for personal protection and bystander intervention.

Initially only developed for grades 1–6, with subsequent additions for grades 7–12, in 2014–2015, Childhelp initiated a collaboration with the Southwest Interdisciplinary Research Center (SIRC) at Arizona State University to (1) restructure the SUBS curriculum to be consistent across all grades, (2) enhance the lessons using developmentally and educationally appropriate strategies, (3) develop facilitator modules, and (4) position SUBS for efficacy testing. Prior to the SUBS randomized controlled trial (RCT), a pilot study was conducted during the fall and spring of 2016–2017 [34] and included pre- and post-surveys for grades pre-kindergarten to 12 (n = 2424) in addition to implementation feedback from 15 facilitator/social worker surveys. The goal was to pilot all parts of the research design, implementation plan, and schedule to ensure the future success of the RCT. The outcomes were based on comparing pre- and post-data. During the design phase of the measures, survey questions and survey administration protocols were presented to a school district’s social workers, who served as the facilitators. They provided valuable input on the procedures, permission/opt-out forms, surveys, training, lessons, and data collection from which improvements were made. In addition, following the implementation of the pilot, facilitator focus groups were conducted. These qualitative data added insight into what worked well and what lesson segments needed modification.

### 1.2. Present Study—SUBS RCT

The aim of this RCT was to examine the efficacy of the Childhelp SUBS curriculum in a low SES community with a multicultural sample of students across age, grade, and ethnicity. The findings of this study add to the literature by providing the results and implementation data focusing on whether this intervention resonated with a multicultural population and can truly be used as intended for a universal audience. This study builds upon much-needed conversation for creating and implementing an evidence-based universal prevention curriculum appropriate for students at all grade levels that is culturally responsive, effective, and sustainable. While the SUBS lessons are designed for grades pre-kindergarten to 12, this study was specific to an elementary school district with grades kindergarten to 8; a high school district participated in a separate study the following year [35].

## 2. Materials and Methods

### 2.1. Sample

The majority of the participants were racial and ethnic minority students (90%) and qualified for the free/reduced lunch program (80%). The participants included students in kindergarten through grade 8. The students in the schools assigned to the SUBS intervention condition received the curriculum, and the students who attended schools assigned to the control condition did not receive the program. The students in both the SUBS and control schools completed all three surveys. All the schools (SUBS and control) were offered the curriculum free of charge the next school year. There was a total of 2797 participants (SUBS = 1573; control = 1224) with matched pre-, post-, and follow-up surveys. The demographic data were collected for each grade, as were the gender and age data; the ethnicity data were collected in grades 6 through 8 (see Table 1).

### 2.2. Procedures

#### 2.2.1. Schools and Randomization

In the district, the schools were randomly assigned to either the SUBS or control condition. Block randomization occurred by first creating two alphabetized school lists based on school size (500 or more students; 499 or fewer students). A coin toss was used to assign the schools alternately to each condition based on their size. Initially, eight schools were assigned to the SUBS condition, and seven schools were assigned to the control condition. However, before implementing the program, one principal withdrew their school from the study (control condition), and after two months, an implementation school withdrew, as the social worker assigned to the school resigned, leaving thirteen schools: seven implementation schools and six control schools.

#### 2.2.2. Measures

The measures were designed to assess the efficacy of the SUBS curriculum in increasing and retaining children’s knowledge of safety rules and self-protection/resistance (RESIST) strategies. The survey items were developed based on the learning objectives and key terms taught within each grade level. A readability analysis was conducted for each item, using the Flesch–Kincaid Grade Level score, which is a widely used readability formula that assesses the approximate reading grade level of a text [41], to ensure age appropriateness. The survey questions were piloted and revised before the randomized controlled trial. The content validity of the measures was addressed in the pilot study [34].

The survey items differed depending on grade level. In kindergarten through grade 2, pre-surveys included three knowledge items regarding safety issues as well as demographic items. For example, one item was, *do you think it is a grown-up’s job to keep you safe?* The surveys for kindergarten through grade 2 were administered orally. In grades 3 through 8, the student surveys included multiple-choice items about each safety rule, such as *Safety Rule #1: It’s ___ Body!* In grades 4 through 8, the students were additionally asked questions about the RESIST strategies, for instance, *what does R mean?* The same questions were asked for each grade level on the pre-, post-, and follow-up surveys, with additional learning-assessment-related items only on the post- and follow-up questionnaires. Demographic questions were asked only on the pre-surveys.

#### 2.2.3. Procedure and Implementation Protocol

All the protocols and instruments for data collection were reviewed and approved by the Social Behavioral Institutional Review Board at Arizona State University. Before starting the implementation of the curriculum, the schools’ social workers completed an online facilitator training, which included universal modules on child abuse and neglect and modules specific to the lessons for the grade levels they were facilitating. The opt-out forms went home to parents, letting them know that their child would participate in a research study, and gave information on how to opt their child out of the surveys. The social workers collected the opt-out forms with only a handful of students in each school who did not participate in the study because their parents opted them out of the lessons. The SUBS and control condition participants were required to indicate their assent to participate before completing the surveys. The youth assent forms were attached to each study for grades 3 through 8 and were read to the students in K through 2; youth who did not wish to participate were asked to accompany a teacher to a different location for the survey duration. For fidelity purposes, SIRC researchers conducted lesson and facilitator observations. The social workers also participated in a 1.5 h training on tracking opt-out forms, assigning student codes, and conducting pre-, post-, and follow-up surveys.

#### 2.2.4. Data Collection

The pre- and post-surveys were administered in fall 2018, with approximately six weeks between pre- and post-survey completion; the students completed the pre-survey before receiving their first lesson and the post-survey following their second and final lesson. About six months later (late spring 2019), the follow-up survey was administered. All these three surveys were administered on paper. Each student was assigned a unique identification number, and the facilitator provided the sticker with that number to each student to adhere to their survey at all three survey administration times. The unique identification number was used to match the three surveys to each student and only those with the three surveys were included as respondents. The data files were created in SPSS, and the survey data were entered and cleaned for analysis. To account for possible challenges with the data, the following measures were put into place to ensure the data were accurate and reliable: hiring and training data entry clerks, coding missing values appropriately, and performing data quality assurance checks.

#### 2.2.5. Data Analysis

The demographic information was examined and reported based on the pre-survey data. Safety knowledge, safety rules, and RESIST strategy score differences were examined at follow-up for comparisons between the SUBS and control participants. Within each grade level, for each question, responses to items were scored correct (1) and incorrect (0). A cumulative knowledge score was calculated for each student, for each index or RESIST scale, by adding the number of correct answers. Next, a correct percentage out of 100% was generated for each student for each index or RESIST scale. In grades kindergarten through 2, there were three general safety knowledge items; these were examined as an index. In grades 3 through 8, there were five items specific to the curriculum’s safety rules, and these five were examined as an index. In grades 4 through 8, there were six resistance strategy items; principal component analyses were conducted for the six resistance strategy items for each grade, the results of which suggested the validity of the six-item RESIST scale. The internal consistency was examined using Cronbach’s alpha within each grade. Each alpha value was acceptable and ranged from 0.76 to 0.82 across the grades [36].

The analyses explored the differences between the pre- and follow-up surveys for the general safety knowledge index, the safety rules index, and the RESIST scale. The mean differences in these constructs between the individuals in the SUBS and control groups were separately analyzed for each grade using the analyses of covariance (ANCOVA) that included the pre-survey scores as a covariate.

## 3. Results

### 3.1. Safety Knowledge

The students in kindergarten, grade 1, and grade 2 were asked a series of questions about safety knowledge. These safety knowledge questions were examined as a safety knowledge index. The analysis compared the follow-up scores on the safety knowledge index for each grade separately using the analysis of covariance to control for the pre-survey scores. In kindergarten through grade 2, the SUBS students scored significantly higher at follow-up on the safety knowledge index than the students in the control group after controlling for the pre-survey safety knowledge scores: kindergarten, F (1, 237) = 5.985, *p* = 0.015; grade 1, F (1, 252) = 14.959, *p* =0.000; grade 2, F (1, 326) = 7.761, *p* = 0.006. The partial eta-squared effect sizes ranged from small to medium (see Table 2).

### 3.2. Safety Rules

The participants in grades 3 through 8 were asked a series of questions related to the safety rules taught in the SUBS curriculum. The students in grades 3, 4, 5, and 6 who participated in the SUBS curriculum scored significantly higher at follow-up on the safety rule index than the students in the control group after controlling for the pre-survey safety rule scores: grade 3, F(1, 203) = 13.183, *p* = 0.000; grade 4, F(1, 390) = 28.512, *p* = 0.000; grade 5, F(1, 418) = 32.090, *p* = 0.000; grade 6, F(1, 340) = 36.699, *p* = 0.000. The SUBS students in grade 7, F(1, 231) = 0.430, *p* = 0.513, and grade 8, F(1, 239) = 2.340, *p* = 0.127, did not score significantly higher at follow-up on the safety rule index than the students in the control group. The partial eta-squared effect sizes ranged from small to medium (see Table 3).

### 3.3. RESIST Strategies

The students in grades 4 through 8 were asked about the various RESIST strategies taught in the SUBS curriculum. The students in grades 4, 5, 6, and 8 who participated in the SUBS curriculum scored significantly higher at follow-up on the RESIST scale than the students in the control group after controlling for the pre-survey RESIST strategy scores: grade 4, F(1, 388) = 29.282, *p* = 0.000; grade 5, F(1, 407) = 56.498, *p* = 0.000; grade 6, F(1, 344) = 7.329, *p* = 0.007; grade 8 F(1, 243) = 11.909, *p* = 0.001. Grade 7 students who participated in the SUBS curriculum did not score significantly higher at follow-up on the RESIST scale than the students in the control group, F(1, 236) = 1.682, *p* = 0.196. The partial eta-squared effect sizes ranged from small to medium (see Table 4).

### 3.4. Learning Assessment

All the SUBS intervention group students’ learning assessment data were collected from the post- and follow-up surveys. Within each grade, the number of these items varied. For example, there were three items in kindergarten, whereas, in grade 8, there were eight items. Overall, the SUBS participants reported they acquired new learning on CM-related safety from these lessons. Over 80% of the children in kindergarten through grade 2 reported learning new safety rules because of the program; additionally, over 74% of the participants in grades 3 through 8 shared that they learned new ways to keep themselves safe after participating in the program. Further, when the students in grades 3 through 8 were asked, the majority of the participants indicated they were more prepared to speak up in an unsafe situation.

## 4. Discussion

This randomized controlled trial showed positive results for the students (K-8) who participated in the SUBS curriculum compared with those in the control group, who did not receive the lessons. The SUBS students learned general safety knowledge, safety rules, and RESIST strategies taught in the lessons and demonstrated this knowledge at follow-up. There were significant findings for most of the items across the grades, indicating that these lessons yield effective results when taught to a multicultural population with a majority of racial and ethnic minority students in a low SES area.

The study design included the methods suggested in the literature and clearinghouses to advance the field of prevention programs and evidence-based practices. One of the primary strengths of this randomized controlled trial was the ability to test the efficacy of the lessons with a robust matched sample of students that included pre-, post- and follow-up surveys in the same school year. The SUBS randomized controlled trial met rigorous design requirements and showed significant evidence of effectiveness at the majority of the grade levels across 11 of the 14 measured indexes/scales.

It was especially encouraging that the results were positive across the three measures studied: safety knowledge, safety rules, and RESIST strategies. For the 14 measures across the 3 sets of indexes/scales and 9 grade levels, the SUBS group had significantly higher scores at follow-up than the students in the control group for the majority of these outcomes. Further, when asked to assess their learning, 7 of 10 students across all the grades reported they learned new safety rules (K-2) or learned new ways to keep safe (the specific question varied by grade).

These findings are important to demonstrate that students from kindergarten through grade 8 can learn knowledge to help them be safe and know when unsafe behaviors around child abuse and neglect may be happening to them or others. For the young students in kindergarten and grades 1 and 2, these results showed they could understand the safety knowledge around child maltreatment and begin to learn why personal safety is important. The five safety rules taught in the SUBS lessons provided students in grades 3 through 8, significantly for those in grades 3, 4, 5, and 6, with the safety skills that they can use in all situations to protect themselves physically and emotionally and be helpful to others in need. The activities used during the lessons presented these five safety rules multiple times in easy-to-remember ways, enabling the students, as shown in these results, to retain this information and gain prevention and intervention skills. Overall, the students indicated that they learned new ways to keep themselves safe.

The RESIST strategies taught to grades 4 through 8 showed significant positive findings in the students in grades 4, 5, 6, and 8, providing them the recognition to combat CM. The positive results for all the grades showed that students learned multiple strategies to RESIST child abuse and neglect, allowing them to feel empowered in frightful situations and know they have options to prevent or interrupt future abuse. These are valuable life skills that result from participation in the SUBS curriculum.

The study’s findings are consistent with the literature demonstrating there are significant learning gains for children, even young children, who participate in school-based victimization prevention programs [30]. This curriculum provides the information, knowledge, and skills necessary for students to take action, i.e., to act using specific behaviors that address child abuse and neglect for that individual or to help others as a bystander to these behaviors. By gaining an understanding and knowledge of CM, young and adolescent students can develop skills to recognize these malicious acts and know the strategies that allow them to speak up and be safe for themselves and others. Thus, this study demonstrated the SUBS curriculum to be effective and provides educators, prevention scientists, child welfare advocates, and policymakers with one more vital tool to address CM with those who are most vulnerable.

The use of effective CM programs holds the promise that the risk factors and the negative health consequences that follow can be mitigated, having positive impacts on communities, schools, families, and individuals. To date, the SUBS curriculum showed positive results with a universal group of racially and ethnically diverse low-SES students in an urban area. Additional studies with students in other states or countries are warranted, especially in light of the worldwide nature of the CM public health crisis, and the already-in-place use of SUBS across the U.S. and internationally. Further, while schools provide an easily accessible and cost-effective means to provide prevention strategies to many children simultaneously, SUBS may also be a useful tool within community-based settings to help youth avoid the physical, behavioral, and psychological problems that result from CM.

### Limitations

This randomized controlled trial has a few limitations. First, the results relied on self-reported data from elementary and middle school students. Whether or not all the students completely understood the questions on the survey is unknown. It is possible the students may have responded dishonestly or with socially desirable answers, which can be a potential threat to internal validity. The facilitators assisted those students who could not read. Those students with disabilities or English as a second language were not included in the sample. Second, the researchers could have conducted, or could as future research conduct, a qualitative follow-up component such as focus groups with youth to help measure the specific knowledge and skills they acquired from these lessons. Lastly, the pilot study before the randomized controlled trial did not receive survey responses for grade 7, which resulted in a lack of feedback on the curriculum and survey measures for that grade and may have contributed to the lack of significant findings for that grade level.

## 5. Conclusions

The SUBS RCT advances best practices and adds the *Childhelp Speak Up Be Safe* curriculum as an evidence-based program to a short list of K-8 school-based CM prevention programs. The lessons’ knowledge, strategies, and skills are appropriate for students’ grade level and developmental period. This study was purposefully designed to utilize a universal sample of multicultural students from kindergarten to grade 8 and to have the lessons delivered by trained social workers who were already assigned to these schools; both of these are unique design elements that make this research real-world-based and applicable to many schools and districts. When randomized control was used to test incorporating the pre-, post-, and follow-up surveys, this program produced significant positive outcomes for multiple grades and measures, showing that the students learned new knowledge and gained the skills to act upon that knowledge to identify child maltreatment and keep themselves and others safe.

Important to CM prevention programs, SUBS teaches the essential concepts of child abuse and neglect for students to recognize and prevent CM and contributes to a lessening of child abuse, neglect, and bullying. With CM identified as an international public health concern, exacerbated by the COVID-19 pandemic, evidence-based universal CM prevention programs are now needed more than ever to address this public health crisis. Policy- and decision makers, as well as educators and parents, now know that as an effective universal CM prevention program, the SUBS curriculum can be implemented in diverse schools and settings at a low cost, delivering an essential safety program that protects students from child abuse and neglect.

## Figures and Tables

**Table 1:** Demographics.

	Total	SUBS	Control
Variable	*n* (%)	*n* (%)	*n* (%)
School	13 (100%)	7 (53.8%)	6 (46.2%)
Free/Reduced Lunch	2539 (100%)	1415 (55.7%)	1124 (44.3%)
Grade Characteristics			
**Kindergarten**	258	158	100
School	11	6	5
Boy	124 (48.1%)	83 (52.5%)	41 (41.0%)
Girl	127 (49.2%)	70 (44.3%)	57 (57.0%)
Unknown	7 (2.7%)	5 (3.2%)	2 (2.0%)
**First**	272	175	97
School	11	7	4
Boy	132 (48.5%)	88 (50.3%)	44 (45.4%)
Girl	136 (50.0%)	83 (47.4%)	53 (54.6%)
Unknown	4 (1.5%)	4 (2.3%)	0 (0%)
**Second**	340	228	112
School	11	6	5
Boy	158 (46.5%)	105 (46.1%)	53 (47.3%)
Girl	180 (52.9%)	121 (53.1%)	59 (52.7%)
Unknown	2 (0.6%)	2 (0.9%)	0 (0%)
**Third**	222	112	110
School	11	6	5
Boy	112 (50.5%)	57 (50.9%)	55 (50.0%)
Girl	110 (49.5%)	55 (49.1%)	55 (50.0%)
Unknown	0 (0%)	0 (0%)	0 (0%)
**Fourth**	416	264	152
School	11	6	5
Boy	211 (50.7%)	132 (50.0%)	79 (52.0%)
Girl	205 (49.3%)	132 (50.0%)	73 (48.0%)
Unknown	0 (0%)	0 (0%)	0 (0%)
**Fifth**	442	222	220
School	12	6	6
Boy	215 (48.6%)	109 (49.1%)	106 (48.2%)
Girl	221 (50.0%)	111 (50.0%)	110 (50.0%)
Unknown	6 (1.4%)	2 (0.9%)	4 (1.8%)
**Sixth**	357	203	154
School	12	6	6
Boy	174 (48.7%)	96 (47.3%)	78 (50.6%)
Girl	181 (50.7%)	106 (52.2%)	75 (48.7%)
Unknown	2 (0.6%)	1 (0.5%)	1 (0.6%)
White	18 (5.0%)	13 (6.4%)	5 (3.3%)
Hispanic	337 (94.4%)	190 (93.6%)	147 (95.9%)
Black	39 (10.9%)	23 (11.3%)	16 (10.5%)
AI/AN	21 (5.9%)	12 (5.9%)	9 (5.9%)
Asian/PI	11 (3.1%)	8 (3.9%)	3 (2.0%)
Other	2 (0.6%)	0 (0%)	2 (1.3%)
**Seventh**	241	94	147
School	11	5	6
Boy	126 (52.3%)	47 (50.5%)	79 (54.1%)
Girl	110 (45.6%)	45 (48.4%)	65 (44.5%)
Unknown	5 (2.1%)	2 (1.1%)	3 (1.4%)
White	7 (2.9%)	5 (5.3%)	2 (1.4%)
Hispanic	224 (92.9%)	85 (90.4%)	139 (94.6%)
Black	27 (11.2%)	16 (17.0%)	11 (7.5%)
AI/AN	14 (5.8%)	8 (8.5%)	6 (4.1%)
Asian/PI	7 (2.9%)	5 (5.3%)	2 (1.4%)
Other	3 (1.2%)	1 (1.1%)	2 (1.4%)
**Eighth**	249	117	132
School	10	4	6
Boy	134 (53.8%)	69 (59.0%)	65 (49.6%)
Girl	110 (44.2%)	47 (40.2%)	63 (48.1%)
Unknown	5 (2.0%)	1 (0.9%)	4 (2.3%)
White	8 (3.2%)	3 (2.6%)	5 (3.8%)
Hispanic	241 (96.8%)	103 (88.0%)	138 (104.5%)
Black	36 (14.5%)	19 (16.2%)	17 (12.9%)
AI/AN	10 (4.0%)	5 (4.3%)	5 (3.8%)
Asian/PI	3 (1.2%)	1 (0.9%)	2 (1.4%)
Other	3 (1.2%)	1 (0.9%)	2 (1.4%)

Note. Hispanic denotes participants who identified as Hispanic, Mexican, Mexican American, or Chicano. Race/Ethnicity categories do not add up to 100 percent due to more than one category that could have been selected.

**Table 2:** Means, standard deviations, and ANCOVA statistics for safety knowledge.

Grade	SUBS		Control		ANCOVA		
	M	SD	M	SD	F ratio	Df	η^2^*_p_*
Kindergarten	0.84	0.22	0.77	0.26	5.985 *	1, 237	0.025
1	0.85	0.21	0.72	0.28	14.959 ***	1, 252	0.056
2	0.84	0.23	0.77	0.23	7.761 **	1, 326	0.023

* *p* < 0.05. ** *p* < 0.01. *** *p* < 0.001.

**Table 3:** Means, standard deviations, and ANCOVA statistics for safety rules.

Grade	SUBS		Control		ANCOVA		
	M	SD	M	SD	F Ratio	Df	η^2^*_p_*
3	0.91	0.18	0.81	0.19	13.183 ***	1, 203	0.061
4	0.95	0.12	0.87	0.19	28.512 ***	1, 390	0.068
5	0.96	0.13	0.87	0.17	32.090 ***	1, 418	0.071
6	0.98	0.07	0.89	0.17	36.699 ***	1, 340	0.097
7	0.92	0.20	0.89	0.17	0.430	1, 231	0.002
8	0.95	0.14	0.91	0.17	2.340	1, 239	0.010

*** *p* < 0.001.

**Table 4:** Means, standard deviations, and ANCOVA statistics for RESIST strategies.

Grade	SUBS		Control		ANCOVA		
	M	SD	M	SD	F Ratio	Df	η^2^*_p_*
4	0.94	0.16	0.82	0.28	29.282 ***	1, 388	0.070
5	0.96	0.17	0.77	0.29	56.498 ***	1, 407	0.122
6	0.94	0.16	0.89	0.21	7.329 **	1, 344	0.021
7	0.94	0.20	0.91	0.20	1.682	1, 236	0.007
8	0.97	0.14	0.89	0.22	11.909 **	1, 243	0.047

** *p* < 0.01. *** *p* < 0.001.

## Data Availability

Data are not publicly available.
